# A lathe system for micrometre-sized cylindrical sample preparation at room and cryogenic temperatures

**DOI:** 10.1107/S1600577519017028

**Published:** 2020-01-29

**Authors:** Mirko Holler, Johannes Ihli, Esther H. R. Tsai, Fabio Nudelman, Mariana Verezhak, Wilma D. J. van de Berg, Sarah H. Shahmoradian

**Affiliations:** a Photon Science Division, Paul Scherrer Institut, Forschungsstrasse 111, 5232 Villigen PSI, Switzerland; b Center for Functional Nanomaterials, Brookhaven National Laboratory, New York, NY 11973, USA; c School of Chemistry, University of Edinburgh, David Brewster Road, Edinburgh EH9 3FJ, UK; d Section Clinical Neuroanatomy and Biobanking (CNAB), Department of Anatomy and Neurosciences, Amsterdam UMC, VU University Medical Center, De Boelelaan 1108, Amsterdam 1007, The Netherlands

**Keywords:** Sample preparation, nano-tomography, ptychography

## Abstract

A simple two-spindle based lathe system for the preparation of cylindrical samples intended for X-ray tomography is presented. The setup can operate at room temperature as well as under cryogenic conditions, allowing the preparation of samples down to 20 and 50 µm in diameter, respectively, within minutes. Case studies are presented involving the preparation of a brittle biomineral brachiopod shell and cryogenically fixed soft brain tissue, and their examination by means of ptychographic X-ray computed tomography reveals the preparation method to be mainly free from causing artefacts. Since this lathe system easily yields near-cylindrical samples ideal for tomography, a usage for a wide variety of otherwise challenging specimens is anticipated, in addition to potential use as a time- and cost-saving tool prior to focused ion-beam milling. Fast sample preparation becomes especially important in relation to shorter measurement times expected in next-generation synchrotron sources.

## Abstract 2

A device for mechanical milling of samples for nano-tomography is presented.

## 1. Introduction   

Microscopy allows us to peer into the structural makeup of functional matter (Holt *et al.*, 2013 ▸). Depending on the type of microscopy and incident wavelength (*e.g.* visible light, X-rays or electrons), different sample preparation techniques are needed to enable the measurement and extract the desired information.

Many microscopy techniques can work with thin sections of material and some methods even require such thinning. This can be very beneficial if the features of interest are on a similar length scale as the section thickness, because the images can be easily interpreted. However, sectioning is destructive and the sample volume can be destroyed during sample preparation. By increasing the sample thickness, single projections commonly appear faded due to structural overlap in the third dimension which quickly reaches a level at which interpretation is no longer possible.

In computed tomography (Grangeat, 2010 ▸), this limitation is overcome by reconstructing a 3D image of the specimen by combining many projections of a sample acquired from different unique orientations. To reduce the amount of missing information, thereby achieving the best possible 3D representation, projections should be acquired across an angular range evenly spaced from 0 to 180°. This inherently implies that the sample can be illuminated by the incoming radiation from all directions. Macroscopic samples often have to be shaped and trimmed to dimensions matching the available or desired field of view and/or to achieve a high enough transmission of the radiation through the sample. Although in general all shapes of samples can be examined, cylindrical shapes are preferable as the field of view remains equally filled at every angle and the sample thickness remains constant throughout the tomographic measurement.

In this work, we focus on sample preparation mainly used in X-ray microscopy (Jacobsen, 2019 ▸), especially nano-tomography in the multi-keV energy range. Many existing techniques, such as focused ion-beam milling (Holler, Guizar-Sicairos *et al.*, 2017 ▸; Young & Moore, 2005 ▸), laser microdissection (Nelson *et al.*, 2006 ▸) as well as mechanical cutting using an ultramicrotome (Wanner *et al.*, 2015 ▸; Dykstra, 1992 ▸), can be used to reduce the dimensions of a sample. These techniques have the benefit that a predetermined region can be isolated. Problematic however is the high acquisition and maintenance cost of such equipment as well as the related time-consuming and tedious sample preparation procedures.

It is not always necessary to select a specific region within a sample when there exists a similar structure throughout the entire volume or because there is no possibility to define a region of interest before the sample preparation (Ihli *et al.*, 2017 ▸; da Silva *et al.*, 2015 ▸; Donnelly *et al.*, 2017 ▸; Wilts *et al.*, 2018 ▸). In such cases, the rapid and preferably on-site production of micrometre-scale samples to comply with limitations in both available measurement time and depth of field is desired (Tsai *et al.*, 2017 ▸; Li & Maiden, 2018 ▸). Here, we present a simple two-spindle-based lathe system for the fast and cost-effective preparation of cylindrical samples intended for X-ray tomography. The setup can operate both at room temperature and under cryogenic conditions, allowing the preparation of samples down to diameters of 20 and 50 µm, respectively, within minutes. As a demonstration, we prepared cylindrically shaped pillars of brachiopod shells, a brittle biomineral made of fluorapatite, at room temperature. At cryogenic temperatures, a 50 µm pillar of cryogenically fixed brain tissue was trimmed. Both samples were prepared on OMNY pins (Holler, Raabe *et al.*, 2017 ▸) and were measured at the cSAXS beamline, Paul Scherrer Institut, Switzerland, using ptychographic X-ray computed tomography (PXCT) (Dierolf *et al.*, 2010 ▸; Guizar-Sicairos & Fienup, 2008 ▸) with the OMNY microscope (Holler *et al.*, 2018 ▸).

## 2. Mechanical arrangement of the sample trimmer   

**Figure 1:**
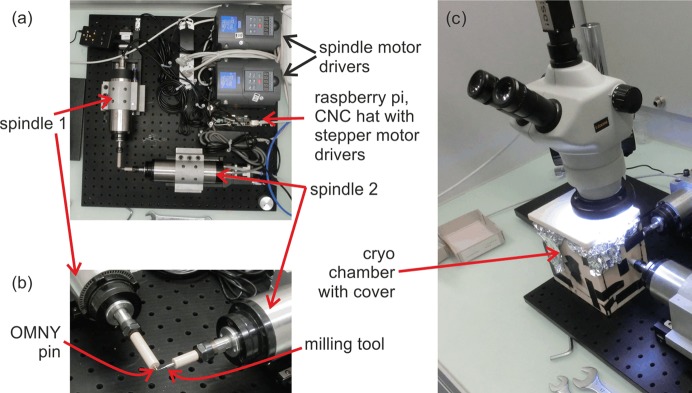
Mechanical arrangement of the sample trimmer. (*a*) Top view of the complete setup. (*b*) Close-up of the milling area. (*c*) First version of a cryogenic chamber and microscope installed.

The sample trimmer is constructed on a breadboard with dimensions of 60 cm × 60 cm [Fig. 1 ▸(*a*)]. Two high-speed engraving/milling spindles (0.8 kW engraving drive, Zhon Jua Jiang, 65 mm diameter × 200 mm length) are mounted on two linear stages (OWIS LTM 60 MSM) and arranged at an angle of 90° to each other. The spindles have a specified runout of less than 5 µm and a maximum rotation frequency of 400 Hz. The measured runout at low frequencies was below 1 µm and mainly dependent on the collet quality used for mounting the tools.

Spindle 1 is air-cooled and operated at 30 Hz. An adaptor was manufactured from polyether ether ketone (PEEK) and clamped to the spindle to allow direct mounting of custom-made ‘OMNY’ tomography pins (Holler, Raabe *et al.*, 2017 ▸), on which the to-be-trimmed sample material is mounted. The adaptor thermally isolates the pin and sample from the spindle, which is important for the preparation of samples under cryogenic conditions.

Spindle 2 operates at its maximum rotation frequency of 400 Hz and is water-cooled. Water cooling and rotation speed were selected to ensure operation stability and to minimize air turbulence otherwise caused by an air-cooling mechanism. Similarly to Spindle 1, an adaptor piece made from PEEK is installed, into which the selected milling tool (here, Brütsch/Rügger Tools, VHM Futuro Type N, 0.5 mm in diameter) snuggly fits. The adaptor isolates the milling tool for operation under cryogenic conditions from the spindle.

Fig. 1 ▸(*b*) shows a close-up of the milling region. Both spindles can move independently on linear stages along their axes of rotation. In the milling process, a large sample is mounted on a sample pin which is then continuously rotated by Spindle 1. Spindle 2 is allowed to approach the sample in predefined incremental steps, gently trimming the sample to the desired cylindrical geometry and diameter by means of repeated surface abrasion.

As depicted in Fig. 1 ▸(*c*), measurements under cryogenic conditions are enabled by utilization of a liquid-nitro­gen bath. To this end, a container constructed from structural foam plates (Airex) and covered with aluminium foil is mounted on the breadboard and partially filled with liquid nitro­gen. By means of portholes, the tips of both spindles are inserted into this container, allowing sample milling to proceed as previously described. The holes are not sealed such that there is a continuous stream of cold, evaporated nitro­gen flowing out. We emphasize that the trimming is not carried out in liquid nitro­gen but slightly above the liquid-nitro­gen level in the cold gas atmosphere. There is enough space in the chamber to allow easy mounting and unmounting of samples as well as keeping them under cryogenic conditions. Moreover, the milling process can be observed by a stereomicroscope from the top.

## 3. Control system   

The control system fits completely onto the breadboard. The main control is performed using a Raspberry Pi 3 (https://www.raspberrypi.org/), to which a CNC hat (http://www.Protoneer.co.nz), GRBL firmware v1.1, is mounted. The latter turns standard machine CNC G-code commands (https://en.wikipedia.org/wiki/G-code) into stepper signals. Two Pololu A4988 stepper drivers are used to control the two linear stages. The drivers are used with 1/16 microstepping, such that one microstep corresponds to 0.3 µm, allowing for smooth and accurate trajectories.

The two spindle motors are controlled using Isacon Power Control A131 spindle drivers. The CNC hat’s coolant enable output is connected to the enable input of Spindle 1, such that the spindle can be activated and deactivated using G-commands. Spindle 1 is set to a fixed rotation speed of 30 Hz. The CNC hat’s spindle enable output (pulse-width modulated) is interfaced to Spindle 2, such that its frequency can be controlled remotely.

The G-code is generated by a simple Python script (van Rossum, 1995 ▸), which uses input parameters for the final sample diameter, sample height, cutting steps and speed. The generated text file is then loaded to the software *bCNC*, which offers a GUI visualization of the process and sends the CNC G-code commands to the CNC hat.

## 4. Example of milling at room temperature   

For a demonstration of the devised setup, a piece of a dry shell from the brachiopod discinisca tenuis (Pérez-Huerta *et al.*, 2007 ▸) was mounted on an OMNY pin (Holler, Raabe *et al.*, 2017 ▸), then milled and imaged using PXCT under cryogenic conditions using the OMNY microscope (Holler *et al.*, 2018 ▸).

**Figure 2:**
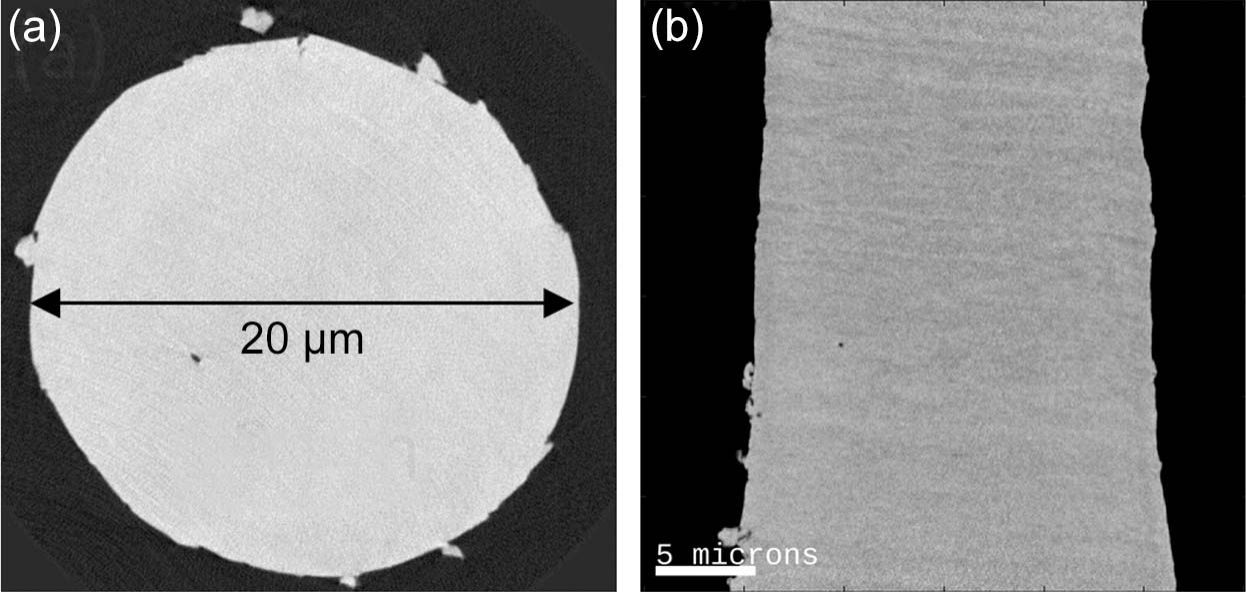
Electron density tomogram of a cylindrical brachiopod shell sample prepared using the lathe system and reconstructed using ptychographic X-ray computed tomography: (*a*) axial and (*b*) sagittal views through the electron density tomogram are shown. PXCT measurements were conducted under cryogenic conditions in OMNY. Voxel size is (38.8 nm)^3^, 3D resolution ≃ 80 nm.

Small shell pieces with radial dimensions in the range 0.5 mm to 1 mm have been manually broken off and have been glued to OMNY pins using Epotek 302 ep­oxy. After curing, the pins were mounted on the sample trimmer and the milling process was started. The resulting pillar had a diameter of 20 µm and a sample height of 50 µm. The active milling time was ∼5 min. Movie S1 of the supporting information shows the milling process. Fig. 2 ▸(*a*) shows an axial cut and Fig. 2 ▸(*b*) shows a sagittal cut though the retrieved electron density tomogram of the prepared pillar. The data reconstruction followed the procedure described by Holler *et al.* (2014 ▸). The 3D spatial resolution estimated using Fourier shell correlation (van Heel & Schatz, 2005 ▸) was 80 nm. While the structure of the shell and cylindrical shape of the pillar are nicely resolved in the tomogram, no cracks or damage inside the sample could be identified within the volume.

The characteristic laminated microstructure of the brachiopod shell is visible, consisting of layers with higher mineral content, which appear brighter, alternating with layers that are richer in organic components such as proteins and polysaccharides, which appear slightly darker (Williams & Cusack, 1999 ▸).

## 5. Example of milling under cryogenic conditions   

For a demonstration of the cryogenic compatibility of the devised setup, an unstained brain tissue sample (see Appendix *A* [App appa]) was mounted on an OMNY pin, milled and then imaged using PXCT. For sample preparation, mounting and sample details, refer to the work by Shahmoradian *et al.* (2017 ▸). The original size of the piece of tissue mounted on the pin was 1 mm in diameter.

**Figure 3:**
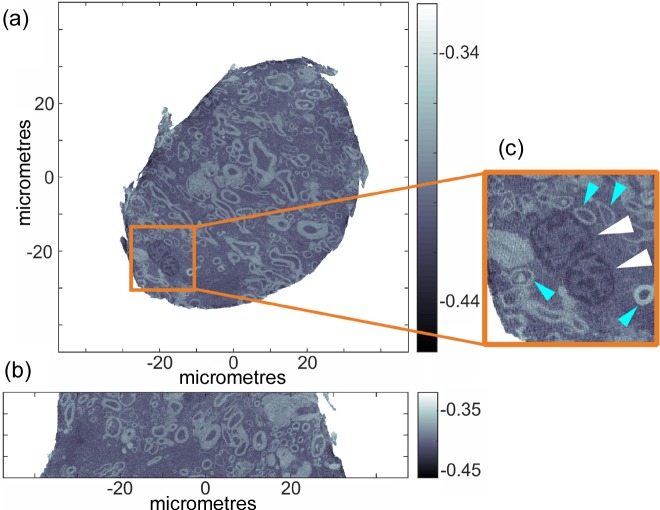
Electron density tomogram of a cylindrical unstained brain tissue sample prepared using the lathe system under cryogenic conditions and measured using ptychographic X-ray computed tomography: (*a*) axial view; (*b*) sagittal view. Variations in grayscale represent changes in electron density (e A^−3^). (*c*) Magnified region of (*a*) showing nuclei with euchromatin (white arrowheads) and axonal cross-sections (blue arrowheads). PXCT measurements were conducted under cryogenic conditions.

For milling under cryogenic conditions, the cryo-chamber was installed and filled with liquid nitro­gen. After approximately 10 min, the thermal drifts of the milling tool and pin holder, which are of the order of 30 µm, stabilized. Before milling a sample, an empty copper pin was mounted and trimmed to re-calibrate the milling diameter and height. After a measurement of the dimensions of the resulting copper pillar with a visible-light microscope, the program parameters were adjusted accordingly and the milling process on the tissue sample was initiated. Here a sample diameter of 70 µm and a sample height of 50 µm were targeted. The active milling time was 3 min. Movie S2 shows a video of the milling process. Shown in Figs. 3 ▸(*a*) and 3(*b*) are orthoslices through the retrieved electron density tomogram. Evident from Fig. 3 ▸ are the desired sample diameter and height.

Samples of similar dimensions and pyramidal morphology using a cryo-ultramicrotome took 4–8 h to prepare (Shahmoradian *et al.*, 2017 ▸). The sharp edges of this pyramidal morphology resulted in artefacts in earlier ptychographic tomography volume reconstructions (Shahmoradian *et al.*, 2017 ▸); however, tomograms of cylindrical shape are free of these artefacts. The improved sample shape and smaller dimensions resulted in an increase in image quality, to the point where nuclei with contained euchromatin and clear membrane borders [Fig. 3 ▸(*c*), white arrowheads] could easily be distinguished without the need of any chemical staining. Neuronal axons of varying thickness and small fine axons less than 2.5 µm in diameter could also clearly be resolved (blue arrowheads). Several cellular nuclei are clearly visible throughout the tomogram as well as clear delineations of cellular axons.

## 6. Imperfections: surface roughness, sample symmetry   

**Figure 4:**
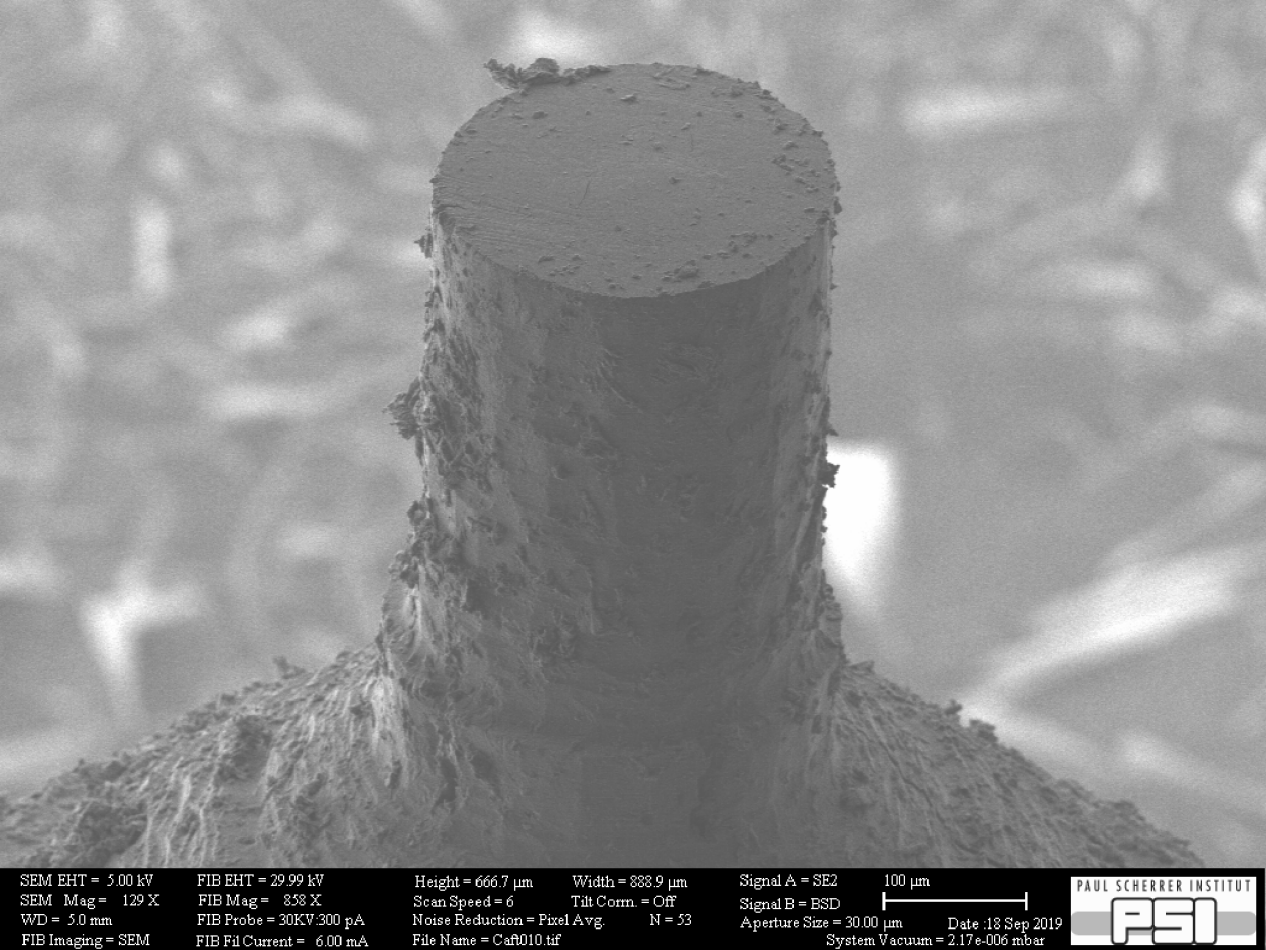
SEM image of a larger sample prepared to a diameter of 280 µm for a measurement via SAXS tensor tomography. The material was cortical bovine bone.

In the aforementioned examples and especially in Fig. 2 ▸, it is apparent that there is a limit to the milling quality that can be reached. Clearly there is some debris present on the surface, meaning that a certain surface roughness remains. While this is absolutely no issue for measuring the sample via PXCT, it could be important for other applications. In particular, we cannot guarantee that the surface is fully intact and undamaged by the milling. While the samples did not develop obvious cracks, such defects may be present close to the surface on a smaller length scale. Fig. 4 ▸ shows an SEM image of a cortical bone sample (see Appendix *B* [App appb]) prepared using the milling tool for a measurement via SAXS tensor tomography (Liebi *et al.*, 2015 ▸). The image allows judgement of the surface quality, and also shows the debris that remains on the surface after milling. Bone was simple to prepare with the milling tool, but is otherwise a difficult material for achieving samples of such dimensions. Diamond knives offer the option to prepare millimetre-sized cubes or thin sections without the possibility to precisely select an area of interest, but these are often problematic in relation to hydration by the saw cooling and cracks generated by knife pressure.

While this larger sample seems to be symmetric and round, an asymmetry appears on the smaller sample in Fig. 2 ▸. This is probably caused by imperfect rotation of the spindle which may be caused by construction and/or a small imbalance. In the case of the tissue sample prepared at cryogenic temperature, it appears that this effect was even larger. In general, trimming at cryogenic temperature seems currently less well controlled compared with trimming at room temperature.

## 7. Summary and conclusions   

In comparison with existing sample preparation methods, such as focused ion-beam milling and laser microdissection, the lathe system presented facilitates easier, faster and cheaper sample preparation of samples in which distinctive nanoscopic features of interest are prevalent throughout the sample, *i.e.* no specific region of interest is pre-determined. Where necessary, the presented system can be used in conjunction with focused ion-beam milling preparation to achieve even smaller and finer cylindrical pillars, since sample sizes even of the order of centimetres can be rapidly trimmed down to dimensions complementary with current focused ion-beam milling instrumentation in minutes. In future, further testing will be carried out with a geometry having both spindles collinear, which may reduce the sensitivity of the setup to thermal drift and hence affect the sample height instead of the sample diameter.

## Supplementary Material

S1: Trimming a 20 micrometre diameter brachiopod shell sample at room temperature. DOI: 10.1107/S1600577519017028/il5047sup1.avi

S2: Trimming a 50 micrometre diameter frozen-hydrated brain sample under cryogenic conditions. DOI: 10.1107/S1600577519017028/il5047sup2.avi

## Appendix A Brain tissue samples

The brain tissue samples were obtained from The Netherlands Brain Bank (NBB, https://www.brainbank.nl). The protocol was approved by the Medical Ethical Committee (METC), VU University Medical Center, Amsterdam, The Netherlands, in compliance with local ethical and legal guidelines, and all protocols were approved by the local institutional review board.

## Appendix B Bone sample

The bone sample was obtained from ‘Berchtold Fleisch AG’. It was extracted from the whole bovine tibia bone from a female cow, 486 days of age. Animal ID: TVD 120.1370.6096.7.
